# Alcohol Septal Ablation or Mavacamten for Obstructive Hypertrophic Cardiomyopathy

**DOI:** 10.3390/jcm12206628

**Published:** 2023-10-19

**Authors:** Smita Scholtz, Volker Rudolph, Jan-Christian Reil

**Affiliations:** Klinik für Allgemeine und Interventionelle Kardiologie/Angiologie, Herz und Diabeteszentrum NRW, Ruhr-Universität Bochum, 32545 Bad Oeynhausen, Germanyjreil@hdz-nrw.de (J.-C.R.)

**Keywords:** hypertrophic cardiomyopathy, left ventricular outflow tract obstruction, septal reduction therapy, alcohol septal ablation, myosin inhibitor, mavacamten

## Abstract

Hypertrophic cardiomyopathy (HCM) is a genetic disease characterized by an increased left ventricular wall thickness in the absence of increased afterload conditions. In addition to diastolic dysfunction, obstruction of the left ventricular outflow tract is common in HCM and has an important influence on symptoms and outcome. Over the last five decades or two decades, respectively, surgical myectomy and alcohol septal ablation were the only therapeutic options if standard medical care was not sufficient. Recently, a new option has become available that has the potential to revolutionize the therapeutic strategies for patients with HCM. Mavacamten is a myosin inhibitor that belongs to a completely new drug class and targets the excessive actin–myosin cross-bridging that is the underlying pathology of HCM. By reducing the actin–myosin interactions, mavacamten not only reduces the left ventricular outflow tract (LVOT) obstruction but also seems to have positive effects on the diastolic function, microcirculation, and cardiac structure. This article summarizes the current evidence on alcohol septal ablation and reviews the preclinical and clinical data on mavacamten for the treatment of patients with obstructive HCM.

## 1. Introduction

Hypertrophic cardiomyopathy (HCM) is a sarcomeric disease that is characterized by increased left ventricular wall thickness in the absence of any apparent clinical reason for hypertrophy, such as increased afterload due to aortic valve stenosis or arterial hypertension. A pathogenic mutation in the sarcomere protein genes can be identified in up to 60% of HCM cases, and the inheritance is autosomal dominant [1]. The most frequent mutations are found in the genes encoding the myosin heavy chain (MYH7) and myosin binding protein C (MYBPC3) [1,2]. In earlier studies, HCM was reported to affect one in 500 people in the general population (0.2%) [3,4,5]. This is probably an underestimate because newer estimates that take into account advanced imaging and genetic testing suggest that the prevalence of HCM in contemporary cohorts is 1 in 200 [6].

**Figure 1:**
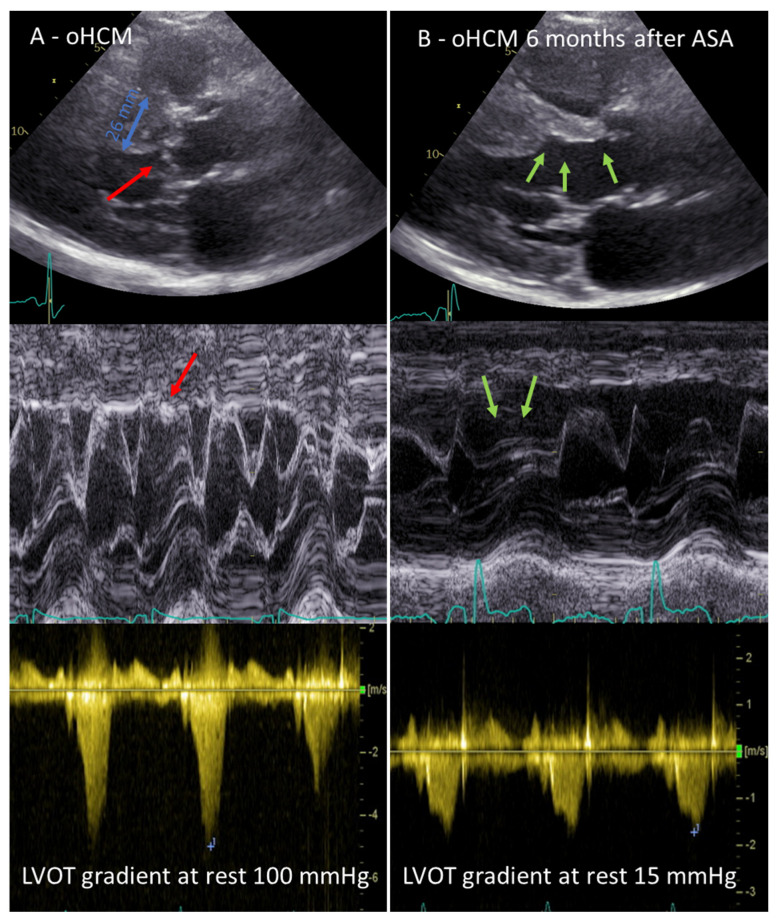
(**A**) Echocardiographic features of obstructive hypertrophic cardiomyopathy (oHCM; septal bulge with maximum diameter of 26 mm; red arrows indicate systolic anterior movement phenomenon of the mitral valve with contact to the septal bulge, left ventricular outflow tract [LVOT] gradient at rest of 100 mmHg); (**B**) Echocardiographic result 6 months after alcohol septal ablation (ASA), green arrows indicate the thinning of the basal septum, resolution of SAM and LVOT gradient at rest of 15 mmHg.

The distribution and severity of hypertrophy can vary greatly. The most common pattern is asymmetrical septum hypertrophy. About 70% of patients have obstructive HCM, with an obstruction of the left ventricular outflow tract (LVOT) or midventricular level at rest or on provocation with the Valsalva manoeuvre or exercise [7]. This dynamic obstruction at the level of the LVOT is caused by septal hypertrophy and the systolic anterior movement (SAM) of the mitral valve leaflets (Figure 1), and is largely dependent on the loading conditions and left ventricular contractility. LVOT obstruction has been shown to be an independent predictor of death related to HCM and the progression of heart failure symptoms [8].

The clinical presentation of patients with HCM also varies widely, from symptom-free or mildly symptomatic to severely symptomatic. Symptoms include dyspnoea, exercise intolerance, angina, syncope, or even sudden cardiac death, and are generally caused by a combination of diastolic dysfunction, microvascular dysfunction and (in patients with obstructive HCM [oHCM]) are mainly due to an LVOT obstruction with associated mitral regurgitation secondary to the SAM phenomenon. Patients with an LVOT gradient ≥ 50 mmHg at rest or with provocation are likely to be symptomatic.

Non-vasodilating β-blockers are currently the first choice of medical therapy for HCM and should be titrated to effectiveness or maximally tolerated dosages. For patients for whom β-blockers are not effective or are poorly tolerated, substitution with a non-dihydropyridine calcium channel blocker (e.g., verapamil or diltiazem) or disopyramide, titrated to the maximum tolerated dose, is recommended [9,10]. If symptoms persist despite optimal medical therapy, septal reduction therapy (SRT) is recommended [9,11].

One method of SRT is surgical myectomy, which was first performed early after HCM disease was first described by Morrow [12] in the 1960s. This technique, referred to as the “Morrow procedure”, is still considered the “gold standard” especially in the United States. The basic steps of this approach include a full sternotomy, cardiopulmonary bypass, and transaortic resection of the hypertrophied myocardium from the proximal anterior ventricular septum under direct visualization. Nowadays, a more extended myectomy is performed (as first described by Messmer [13,14]) and combined with repair/remodelling of the papillary muscles and mitral valve if necessary. With over 50 years’ clinical experience and evidence, surgical myectomy has a class IB recommendation for therapy in severely symptomatic patients with oHCM despite medical treatment according to the 2020 American Heart Association/American College of Cardiology (AHA/ACC) guidelines [9]. However, surgery needs to be performed at specialized HCM centers with high levels of expertise, because outcome and mortality data are significantly better in high volume septal myectomy (SM) centers [15].

Alcohol septal ablation (ASA) is another option for septal reduction therapy, which has a class IC recommendation in the 2020 AHA/ACC guidelines for use in symptomatic adults with oHCM with high surgical risk or are inoperable [9]. However, the situation is quite different in Europe, where ASA has become the first choice of SRT in many HCM centers. Surgery is recommended when oHCM is associated with morphologic abnormalities such as anomalous papillary muscles, markedly elongated mitral valve leaflets, or massive left ventricular hypertrophy, or is accompanied by other lesions requiring surgery [11]. There are no randomized trials evaluating surgical myectomy versus alcohol septal ablation, but several observation studies, reviews, and meta-analyses show that the two procedures have a similar safety and efficacy [16,17].

The myosin inhibitor mavacamten now provides a third option to treat LVOT obstruction in patients with HCM. This novel first-in-class drug specifically targets the underlying pathology of HCM by normalizing myosin and actin cross-bridging to decrease contractility. The data from two phase 3 trials support the efficacy and safety of mavacamten for proving durable improvement in LVOT gradients. In 2023, the European Society of Cardiology (ESC) guidelines for the management of cardiomyopathies included cardiac myosin inhibitors as a second-line therapy (level of evidence IIa) when optimal medical therapy with β-blockers, calcium channel blockers, and/or disopyramide is ineffective or poorly tolerated [10].

## 2. Alcohol Septal Ablation

ASA is a catheter-based method to treat the LVOT obstruction in patients with symptomatic oHCM despite medical therapy. A reduction in the LVOT gradient is achieved by creating a localized septal infarction, which leads to the thinning of the basal septum and the resolution of the SAM phenomenon. Successful use of this procedure in three cases was first reported in 1995 by Ulrich Sigwart [18]. The rationale for this technique was based on the observation that selective balloon occlusion of the first septal artery led to resolution of the LVOT pressure gradient with a regional reduction in the septal hypercontractility and improvement of the diastolic function [19]. The usefulness of infusing ethanol into a coronary artery was derived from the transcoronary chemical ablation of ventricular tachycardia first described by Brugada in 1989 [20]. Over the past 25 years, percutaneous ASA has gained much popularity due to the fact that it is less invasive, effective and safe, even though it has never been studied in randomized trials.

### 2.1. How to Perform Alcohol Septal Ablation—Procedural Steps

The key to successful ASA is careful screening to select the appropriate candidates and discuss them with an interdisciplinary heart team. Individuals with oHCM who have a septal bulge and SAM-associated LVOT obstruction at the level of the basal septum seem to be the best candidates. Other concomitant pathologies such as aberrant papillary muscle contributing to the LVOT obstruction, primary mitral valve pathologies, or other severe valve disease should first be excluded because these individuals are better served by cardiac surgery. A septal wall thickness of ≥17 mm is widely accepted as the lower limit to avoid ventricular septal perforation.

**Figure 2:**
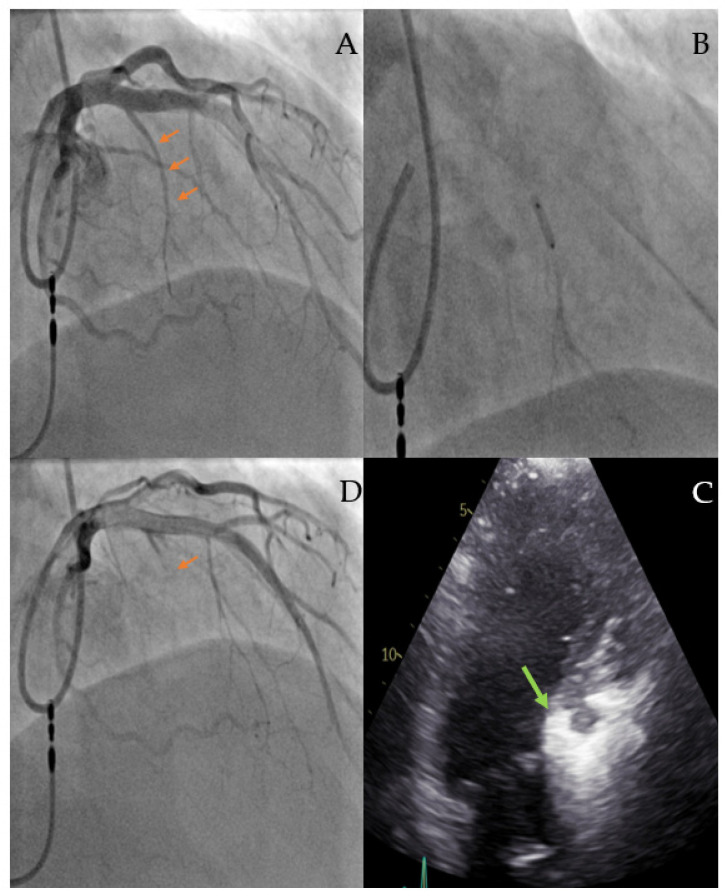
Procedural steps of alcohol septal ablation. (**A**) Angiogram of the left coronary artery (red arrow indicates the first septal branch as the target for ASA); (**B**) Selective angiogram of the first septal branch, which is occluded with an over-the-wire balloon; (**C**) Myocardial contrast echocardiogram verifying the target region (arrows indicate the highlighted septal region); (**D**) Final angiogram of the left anterior descending coronary artery after alcohol injection (arrow indicates the occluded septal branch).

Coronary angiography can be performed at an earlier time or immediately before ASA to rule out concomitant coronary artery disease and to identify the septal branch suitable for alcohol injection. Femoral or radial access are both feasible and the choice is based on the operator’s preference. The standard Judkins left or extra backup guide catheter is engaged in the left coronary ostium. A right anterior oblique cranial view allows for good visualization of the left descending artery with its septal branches. In the majority of cases, the first septal artery originating from the proximal left anterior descending coronary artery (LAD) is the targeted branch; it is rare that the target branch derives from the first diagonal, circumflex, or right coronary artery. Myocardial contrast echocardiography has made an important contribution to the safety of the ASA procedure and was first described by L. Faber in 2004 [21]. After advancing a floppy guidewire and an over-the-wire balloon (OTW) into the target septal branch, the balloon is inflated at a low pressure and the occlusion of the target branch is confirmed by a contrast injection via a guide catheter and selective angiogram of the septal branch via the OTW balloon catheter lumen. This lumen is also used to inject 1–2 mL of an echocardiographic contrast agent (agitated gelantine polysuccinate, Gelafundin 4%, BBraun, Melsungen, Germany) to highlight the myocardial area supplied by the target branch. Simultaneous transthoracic echocardiography in multiple views allows for the verification of the target myocardial area for ASA. In case of contrast displacement (in, for example, the papillary muscle, right ventricular free wall, left ventricular apex, or lateral wall), the occluded septal branch is not suitable for ASA and another septal branch should be tested for suitability. The use of echocardiography to unmask the area supplied by the first septal perforator after contrast dye injection and thus visualization of the presumably infarcted myocardial area due to ASA can be considered the beginning of interventional-guided echocardiography. After confirmation of the correct septal branch by myocardial contrast echocardiography, 95% ethanol is infused very slowly via the balloon catheter lumen. The amount of ethanol is approximately 1 mL for each 10 mm of septal wall thickness. After 10 min, during which time an echocardiographic control with special focus on the wall motion and LVOT gradient is recommended, the OTW balloon can be deflated and retracted from the septal branch. A final angiogram should confirm a patent LAD and an occluded target septal branch (Figure 2).

The continuous hemodynamic monitoring of the LVOT pressure gradient via a second arterial access and placement of an end-hole pigtail catheter in the left ventricle might help to guide the procedure but is not compulsory. Myocardial contrast echocardiography guidance as described above is also feasible.

Before proceeding to ASA, it is necessary to insert a temporary pacemaker lead into the right ventricle via the femoral or jugular vein; this step can be omitted only in patients with an implanted permanent pacemaker or cardioverter/defibrillator. The risk of a permanent complete atrioventricular (AV) block is about 10% and is caused by injury of the conduction system that is located in the immediate vicinity of the myocardial area perfused by the targeted septal branch. A transient AV block is described in up to 30% of cases; therefore, careful monitoring of the heart rhythm is necessary for at least 48 h after ASA to decide whether a pacemaker is indicated. A post-procedural echocardiogram should ideally show an LVOT gradient of <50%; not all patients show an immediate decrease in the LVOT gradient, but may show a decrease a few months after the remodelling of the infarction area [22] (Figure 1).

### 2.2. Clinical Evidence for Alcohol Septal Ablation

There have not yet been any randomized trials comparing the different methods of SRT. Therefore, current therapeutic strategies are based on the data from observational studies, systematic reviews and meta-analyses. ASA has been shown to effectively reduce LVOT obstruction and improve the symptoms, exercise capacity, and oxygen uptake [23,24].

The EuroASA Registry is the largest registry summarizing the long-term outcome data, and includes 1257 patients who underwent an ASA in Europe [25]. The 30-day mortality rate was 1%, and the 1-, 5-, and 10-year survival rates were 98%, 89%, and 77%, respectively. ASA effectively reduced the LVOT gradient from 67 ± 36 to 16 ± 21 mmHg (*p* < 0.01) and the New York Heart Association (NYHA) functional class from 2.9 ± 0.5 to 1.6 ± 0.7 (*p* < 0.01). The post-procedural pacemaker implantation rate was 12%. Another key finding was that higher doses of alcohol were slightly more effective in reducing left ventricular (LV) obstruction but were associated with a higher incidence of peri-procedural complete heart block [25].

Several sub-studies have been published from this registry. In a secondary analysis, a lower dose of alcohol (<2 mL) was associated with higher rates of reintervention compared with higher alcohol doses, without influencing the pacemaker rate [26]. This analysis excluded patients treated with an ultra-low-dose (≤1 mL) and ultra-high-dose (≥3.8 mL) of alcohol because these doses are no longer used. Patients with severe LV hypertrophy ≥ 30 mm had similar procedural outcomes to those with LV hypertrophy < 30 mm, but the long-term all-cause and cardiac mortality rates were higher in the ≥30 mm group [27]. The treatment of mildly symptomatic patients (NYHA II) with a severe LVOT obstruction might have a positive influence on the long-term prognosis, making it comparable to an age- and sex-matched general population; the 30-day mortality rate in this group was very low (0.6%) and the survival rate was 87% [28]. This is clinically relevant because severe LVOT obstruction is associated with a higher risk of heart failure and death [8,29]. Finally, institutional experience was a key determinant of successful outcomes and low complication rates [30]. The all-cause mortality at 30 days was significantly higher in the first 50 consecutive patients treated at each center compared with patients treated later (2.2% vs. 0.5%, respectively). The occurrence of major cardiovascular adverse events was significantly higher in the first group of 50 patients versus the patients that received later procedures (21% vs. 12%; *p* < 0.01), mainly driven by a higher rate of cardiovascular deaths and pacemaker implantation in the patients treated earlier. Even the rates of residual LVOT gradient >30 mmHg and repeated septal reduction therapy were significantly higher in the first 50 versus subsequent patients [30]. Accordingly, the AHA guidelines recommend that ASA be performed in eligible patients at experienced centers (class IC recommendation) [9].

In the absence of randomized trials, several meta-analyses have compared ASA with SM and reported heterogenous results. Osman et al. summarized the data from 4213 patients undergoing ASA and 4240 undergoing SM [17]. ASA was associated with lower periprocedural mortality and stroke (1.2% vs. 2%, *p* = 0.009 and 0.8% vs. 1.5%, *p* = 0.013, respectively) compared with SM, but higher rates of pacemaker implantations and reintervention (10% vs. 5%, *p* < 0.001 and 11% vs. 1.5%, *p* < 0.001, respectively); the long-term all-cause mortality, cardiovascular mortality, and sudden cardiac death rates were similar in both groups [17]. The most recent meta-analyses showed no significant difference in the postoperative all-cause mortality between the patients treated with ASA and SM, but the reduction in the postoperative LVOT pressure gradient and the improvement of the cardiac function were slightly inferior with ASA versus SM, and the risks of pacemaker implantation and reoperation were higher [31,32]. A subgroup analysis of the all-cause mortality based on the data from studies with a follow-up duration of ≥5 years showed a higher long-term mortality with ASA compared with SM [32].

After two decades of use in clinical practice, ASA has been shown to be safe and effective. It has a low complication rate, especially when performed at centers of excellence. However, the weak points are the higher rates of reintervention and pacemaker implantation. Given the patient preference for less invasive procedures, ASA has become the standard procedure for SRT in Europe.

## 3. Mavacamten

Until recently, pharmacotherapy for oHCM was limited to symptom relief via reducing inotropy and the optimizing preload conditions. Non-vasodilating β-blockers were the first-line therapy and, if not tolerated, other pharmacological options were a nondihydropyridine calcium channel blocker or disopyramide (mostly not available in Europe due to a chronic supply shortage).

Mavacamten (formerly MYK-461; Camzyos™) is a novel first-in-class drug that specifically targets the underlying pathology of HCM by normalizing the myosin and actin cross-bridging to decrease the contractility. It was developed by MyoKardia, Inc. (South San Francisco, CA, USA), which is now a wholly owned subsidiary of Bristol Myers Squibb. The US Food and Drug Administration approved mavacamten in April 2022 and in June 2023 it was approved in Europe for the treatment of adults with symptomatic oHCM.

### 3.1. Mechanism of Action

**Figure 3:**
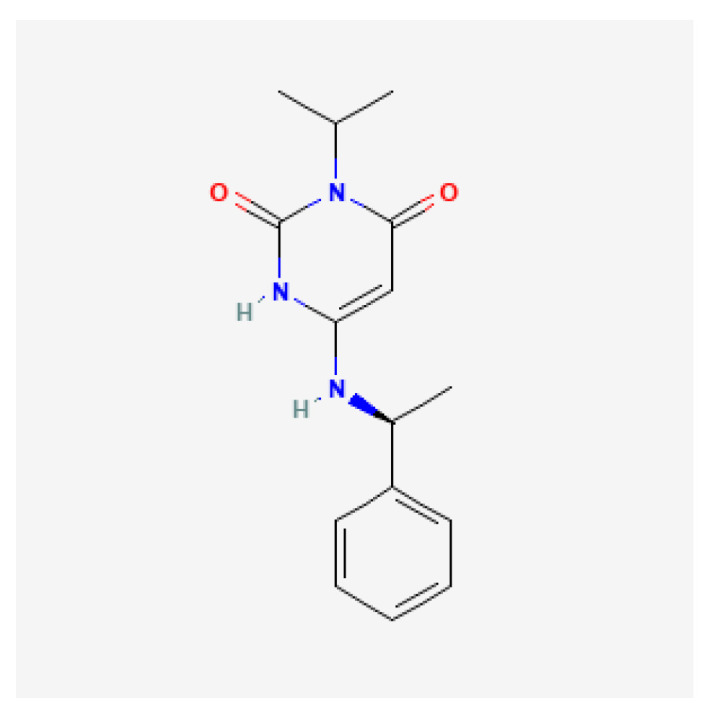
Chemical structure of mavacamten (MYK-461): C15H19N3O2.

**Figure 4:**
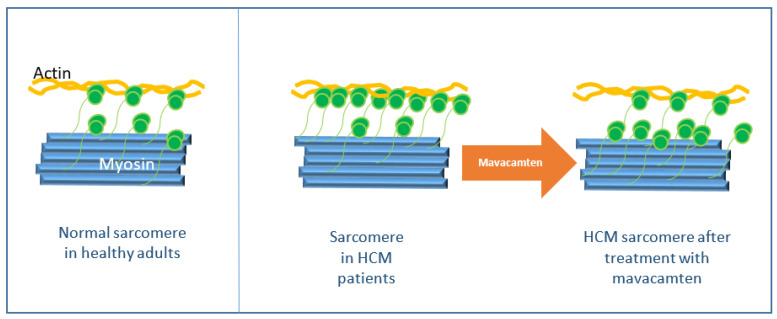
Mechanism of action of mavacamten. Actin–myosin cross-bridges are overexpressed in sarcomeres in individuals with hypertrophic cardiomyopathy (HCM) compared with healthy adults, leading to hypercontractility; mavacamten returns cardiac myosin to a relaxed state.

Mavacamten is a small molecule with the molecular formula C15H19N3O2 (Figure 3). It is an allosteric and reversible inhibitor of the actin-activated cardiac myosin ATPase. Through its binding to myosin ATPase it reduces the number of active myosin heads and thereby reduces the number of actin–myosin cross-bridges. Actin–myosin cross-bridges are overexpressed in patients with HCM, which leads to more ATP consumption and results in energetic insufficiency, hypercontractility, and diastolic dysfunction. Mavacamten brings the cardiac myosin back to a relaxed state and thus optimizes the energy consumption, reduces the hypercontractility and the LVOT gradient, and also improves the diastolic function (Figure 4). The biochemical and molecular nature of the super-relaxed state has been studied intensely because destabilization of this state has been proposed to be a chief cause of HCM. The mechanism of action of mavacamten was shown to be the stabilization of the closed/autoinhibited state of the two-headed cardiac myosin molecule in the thick filament. This forms the basis for modulating the cardiac contractility at the molecular level [33,34,35].

### 3.2. Pre-Clinical Studies

In early 2016, mavacamten was tested in in vitro and in vivo mouse models of HCM to explore whether mutations found in HCM increase the power output at the molecular level. Green et al. showed that the administration of this small-molecule inhibitor of the sarcomere power early in the disease course could attenuate the development and progression of the morphologic, histopathologic, and molecular changes that characterize HCM [36]. These findings support a mechanistic model for HCM in which sarcomere mutations lead to an increased molecular power output, hyperdynamic contraction, and ultimately, pathologic remodelling of the heart [36].

Later in 2016, mavacamten was tested in cats with naturally occurring HCM. The treatment was shown to reduce the contractility, eliminate the systolic anterior motion of the mitral valve, and relieve the LVOT pressure gradient [37]. Prior to the commencement of phase 2 clinical trials, the potential for major drug–drug interactions with mavacamten was excluded, and the drug was found to have a low clearance rate, long half-life, and high oral bioavailability [38].

### 3.3. Clinical Evidence

Table 1 summarizes data from the three main clinical trials of mavacamten that are described below.

PIONEER-HCM (NCT02842242) was an open-label, non-randomized, phase 2 trial that included 21 patients with oHCM from five centers in the United States; the aim was to characterize the effect of mavacamten on the LVOT gradient [39]. The patients were divided into two cohorts: cohort A received mavacamten 10–20 mg/day without background medications and cohort B received mavacamten 2–5 mg/day plus β-blockers at the discretion of the treating physician. The primary outcome was the change in the post-exercise LVOT gradient at the end of the 12-week treatment period. The mean postexercise LVOT gradient decreased from 103 mmHg at baseline to 19 mmHg after 12 weeks of treatment in cohort A (mean change −89.5 mmHg; *p* = 0.008) and from 86 mmHg at baseline to 61 mmHg at week 12 in cohort B (mean change −25.0 mmHg; *p* = 0.020). Mavacamten was well-tolerated and the adverse events were mostly mild (80%) or moderate (19%) in severity. The most common adverse events definitely or possibly related to mavacamten were a decreased left ventricular ejection fraction (LVEF) at higher plasma concentrations (>1000 ng/mL) and atrial fibrillation. In the patients who experienced a reduction in the LVEF to <50%, the withdrawal of the study drug led to the normalization of the LVEF to the baseline status. PIONEER-OLE, a 120-week open-label trial extension, is ongoing and is anticipated to be completed in November 2023.

EXPLORER-HCM (NCT03470545), a multicenter, randomized, double-blinded, placebo-controlled phase 3 trial, was conducted at 68 centers in 13 countries and assessed the efficacy and safety of mavacamten in patients with symptomatic oHCM [40]. A total of 251 patients with oHCM (NYHA class II or III) were enrolled and assigned to treatment with mavacamten or placebo for 30 weeks. Cardiopulmonary exercise testing and post-exercise transthoracic echocardiography were performed at screening and week 30. The serial evaluations every 2–4 weeks included echocardiography, electrocardiography, and blood collection for laboratory tests and determination of the mavacamten plasma concentrations. The primary endpoint was clinical response at week 30, defined as a ≥1.5 mL/kg/min increase in the peak oxygen consumption (pVO_2_) and a reduction in the disease severity of at least one NYHA class, or a ≥3.0 mL/kg/min increase in pVO_2_ without NYHA class worsening. The proportion of patients achieving the primary endpoint was 37% in the mavacamten group versus 17% in the placebo group (*p* = 0·0005). Compared with the placebo group, the mavacamten recipients also had a significantly greater reduction in the post-exercise LVOT gradient (from 86 to 38 mmHg versus from 84 to 73 mmHg). A greater increase in the pVO_2_ and improved symptom scores were also observed in the mavacamten versus the placebo group. A complete response (LVOT gradient <30 mmHg and NYHA class I) was seen in 27% of the patients in the mavacamten group versus 1% in the placebo group. Changes in the baseline systolic function during the treatment with mavacamten were small; the mean reduction in the LVEF was −3.9% versus −0.01% with placebo. The adverse events were generally mild. Serious adverse events were reported by four patients in each treatment group. Two cases of atrial fibrillation and stress cardiomyopathy occurred in the mavacamten group and three cases of atrial fibrillation and one of congestive heart failure occurred in the placebo group. A transient decrease in the LVEF to <50% was observed in seven patients on mavacamten, six of whom had a full recovery after treatment interruption or discontinuation and one patient had partial recovery of the LVEF to 50%. One patient on the placebo experienced sudden cardiac death. The ongoing, long-term extension of this study will provide further evidence for the clinical benefit and safety of mavacamten over 5 years. An interim analysis of 231 patients followed up for 48–84 weeks showed durable improvement in the LVOT gradients, diastolic function, and N-terminal pro B-type natriuretic peptide (NT-proBNP) levels [41].

The analysis of the key echocardiographic parameters assessed by a core laboratory showed significantly improved systolic anterior motion and measures of the diastolic function (a decrease in the left atrial volume index with a mean change from baseline of −7.5 mL/m^2^ and lateral E/e′ with change of −3.8, both *p* < 0.0001) during treatment with mavacamten versus placebo [42]. With these favourable changes in the cardiac structure and function, mavacamten appears to improve the underlying pathophysiology in HCM.

The cardiac magnetic resonance Imaging substudy of the EXPOLORER-HCM trial [43] seems to support these findings on the cardiac structure and function. The patients treated with mavacamten (*n* = 17) had a greater reduction in the mean LV mass index (the primary endpoint) from baseline to week 30 than the patients in the placebo group (*n* = 18; *p* < 0.0001). Furthermore, mavacamten was associated with a greater reduction in the maximum LV wall thickness, intracellular myocardial mass index, and left atrial volume index compared with placebo. However, no changes were observed in the late gadolinium enhancement or myocardial contractile fraction.

VALOR-HCM (NCT04349072) was a phase 3 trial that assessed whether mavacamten would improve the patient symptoms or LVOT gradients enough to defer septal reduction therapy [44]. The study included 112 severely symptomatic patients with oHCM (NYHA III/IV or NYHA II with exertional syncope) with an LVOT gradient ≥ 50 mmHg at rest/provocation who were randomized to mavacamten or placebo. The dose titration from 5 mg/day up to 15 mg/day was based on the LVOT gradient and LVEF. The primary endpoint was eligibility for SRT according to the 2011 guidelines or a patient decision to proceed with SRT. After 16 weeks of treatment, 76.8% of the placebo recipients versus 17.9% of the mavacamten recipients met the guideline criteria or underwent SRT (*p* < 0.001). The difference in the post-exercise LVOT gradient between the mavacamten and placebo groups was −37.2 mm Hg (*p* < 0.001). A functional improvement by at least 1 NYHA class was documented in 62.5% of the patients in the mavacamten group compared with 21.4% of the patients in the placebo group (*p* < 0.001). In addition, changes in the Kansas City Cardiomyopathy questionnaire score, NT-proBNP, and troponin I showed significant differences favouring mavacamten (all *p* < 0.001). Mavacamten was well tolerated; only two patients had a LVEF reduction to <50%, which recovered completely after temporary drug discontinuation. No patients had a reduction in the LVEF to <30% necessitating permanent drug discontinuation. Atrial fibrillation occurred in 3.6% of the patients taking mavacamten and no patients on the placebo. After 16 weeks of therapy, the mavacamten group continued therapy for another 16 weeks and the placebo group crossed over to dose-blinded mavacamten for the same period [45]. There was a sustained reduction in the proportion of patients proceeding to SRT or remaining guideline eligible in the mavacamten group, and benefits were also seen in the patients switched from the placebo. The long-term extension of VALOR-HCM will run for 96 weeks with participants blinded to the dosage of mavacamten followed by an 8-week post-treatment visit. 

### 3.4. Pharmacokinetics and Drug Interactions

Mavacamten is supplied as gelatin capsules for oral use containing 2.5, 5, 10, or 15 mg of mavacamten per capsule. The bioavailability of mavacamten after oral administration is excellent, at 85%. It is rapidly absorbed with a time to maximum concentration of 1 h. Its distribution is through plasma protein binding, which ranges from 97% to 98%. Mavacamten is eliminated by the liver and metabolization is primarily through CYP2C19 (74%), and to a lesser extent by CYP3A4 (18%) and CYP2C9 (8%). Therefore, mavacamten has a variable terminal half-life that depends mainly on the CYP2C19 metabolic status. In CYP2C19 normal metabolizers, the half-life of mavacamten is 6–9 days, which is prolonged to 23 days in CYP2C19 poor metabolizers. This may lead to drug accumulation with increased plasma levels and an increased risk of adverse events [46]. The contraindications and adverse reactions are listed in Table 2.

The inducers and inhibitors of CYP2C19 and moderate to strong inhibitors or inducers of CYP3A4 may affect the systemic exposure to mavacamten. Therefore, the concomitant use of mavacamten with moderate to strong CYP2C19 inhibitors or strong CYP3A4 inhibitors, or with moderate to strong CYP2C19 inducers or moderate to strong CYP3A4 inducers, is contraindicated. Dosage adjustments may be required when mavacamten is given with concomitant administration of weak CYP2C19 inhibitors (e.g., omeprazole) or moderate CYP3A4 inhibitors (e.g., verapamil and diltiazem).

No dosage adjustment is required in patients with mild (Child–Pugh A) to moderate (Child–Pugh B) hepatic impairment, although increased drug exposure is possible. The effects of mavacamten administration in individuals with Child–Pugh C hepatic impairment is unknown.

Due to these variances in the metabolism and variable plasma level, the prescription of mavacamten is strictly regulated. In the United States, it is only available through the Risk Evaluation and Mitigation Strategy (REMS) program with three main aspects (physician–pharmacist–patient) to ensure the safe and monitored administration of the drug. The physician needs to be REMS-certified to be able to prescribe mavacamten, which includes a responsibility to provide accurate counselling. Mavacamten can only be dispensed from certified pharmacies participating in the program and the pharmacist is responsible for reviewing the medication regimens and reinforcing drug interactions and avoidances. Lastly, the patient must commit to regular screening echocardiograms during mavacamten therapy. In Europe/Germany, a further safety aspect has been added before prescribing mavacamten: patients need to be genetically tested for their CYP2C19 metabolism. In CYP2C19 poor metabolizers, the mavacamten starting dosage is 2.5 mg/day and the maximum dosage is 5 mg/day. For all other phenotypes of CYP2C19 metabolizers (intermediate, normal, fast, and ultra-fast), the starting dosage is 5 mg/day and maximum dosage is 15 mg/day. Regular echocardiographic screening is compulsory, with assessment of the LVOT gradient and LVEF every 4 weeks in the first 12 months and every 12 months thereafter. Algorithms for initiation and maintenance dosing, patient monitoring schedules, and guidance for treatment interruption or discontinuation are provided in the prescribing information and should be followed.

## 4. Potential Clinical Use

Since the US and European approvals of mavacamten in April 2022 and June 2023, respectively, there is still a need to define the application of this drug in clinical practice. Neither the European nor the American guidelines for the management of HCM include cardiac myosin inhibitors in the treatment algorithm for HCM. Given that most patients in the randomized trials remained on their background medication with β-blockers or non-dihydropyridine calcium channel blockers, these agents will probably remain the first-line therapy in the near future.

Initially, the prescription of mavacamten is more complicated than prescribing β-blockers due to the need to consider the CYP2C19 metabolic status and have regular echocardiographic follow-up every 4 weeks initially and every 12 weeks thereafter. The annual drug cost for mavacamten was calculated as $US75,000 and the additional cost for regular check-ups may cause issues with the cost effectiveness of this treatment strategy for the health care system. According to the Institute for Clinical and Economic Review, septal reduction procedures dominate mavacamten with respect to cost effectiveness [47].

Furthermore, caution is necessary during treatment with mavacamten because of drug interactions. Regular checks of the medication regimen will be essential to optimize the safety and efficacy during long-term treatment. The concomitant use of strong or moderate CYP2C19 inhibitors with mavacamten is contraindicated because it increases the blood level of mavacamten and may increase the risk of systolic dysfunction and heart failure.

Both phase 3 trials of mavacamten had a follow-up duration of <1 year, therefore the long-term safety of chronic mavacamten therapy, especially regarding the LVEF reduction, is unknown. The data from the long-term extension studies of the randomized trials and long-term post marketing registries will provide more information on this. However, mavacamten is the first drug to show a favourable impact on cardiac remodelling in HCM. Whether it will halt disease progression or even reverse hypertrophy and fibrosis in HCM hearts and its impact on prognosis, long-term outcomes, and mortality remain to be determined.

**Figure 5:**
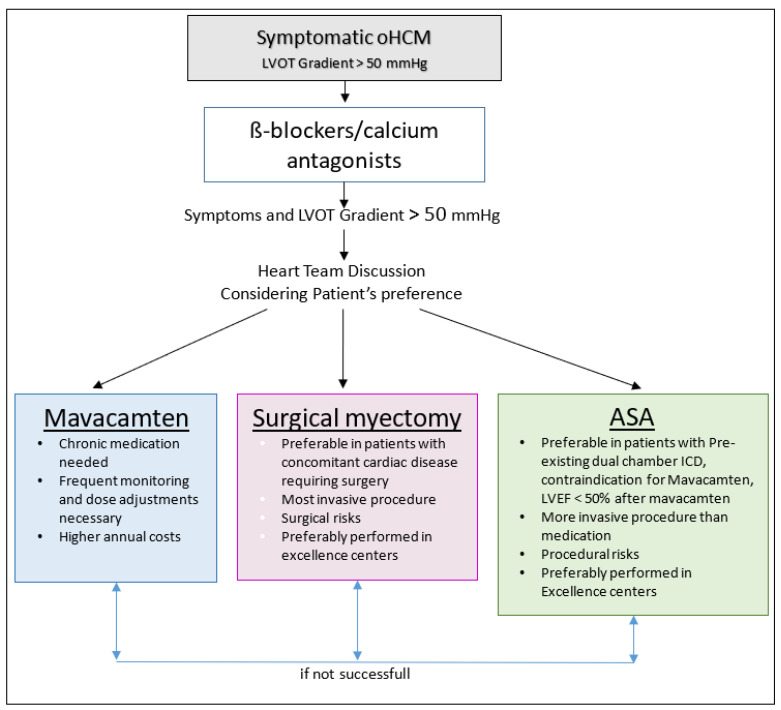
Proposed treatment algorithm for patients with obstructive hypertrophic cardiomyopathy(oHCM). ASA, alcohol septal ablation; ICD, implantable cardioverter defibrillator; LVEF, left ventricular ejection fraction; LVOT, left ventricular outflow tract.

The question of whether mavacamten will replace surgical myectomy or ASA is not quite clear yet. The VALOR-HCM trial showed that mavacamten was effective enough to allow SRT to be deferred. Notably, after the primary study duration of 16 weeks, the vast majority of patients (95%), including 93% of those in the placebo group, chose to continue in this active phase of the trial up to 32 weeks rather than proceed with SRT. This suggests that patients prefer medical therapy over invasive procedures. Clinical experience confirms patients’ preference for medical therapy when they are adequately and extensively informed. However, there were a few patients in all the mavacamten trials who did not respond adequately to the treatment or had side effects that prevented further treatment. Reasons for an unsatisfactory response need to be investigated further (e.g., the type of pathogenetic mutation). These patients will probably still be candidates for SRT. Patients with dual-chamber implantable cardioverter-defibrillators implanted for primary prevention of sudden cardiac death might also be candidates for primary ASA because the major risk of an AV block is already addressed, and other risks of the ASA procedure are negligible. Moreover, it is important to note that SRT is a one-time procedure with a low risk when performed at centers of excellence and is supported by good clinical evidence to eliminate the LVOT gradient and the need for long-term medical therapy. Nevertheless, patients without a suitable septal branch for ASA or who experience reobstruction of the LVOT after any type of SRT now have an alternative medical strategy with mavacamten (Figure 5). Whether HCM patients with midventricular obstruction not related to the SAM phenomenon and hypertrophy of the papillary muscle respond to mavacamten as impressively as patients with the typical SAM-related LVOT obstruction needs to be investigated.

## 5. Future Directions

Both patients and their physicians had high expectations regarding mavacamten. However, the true long-term clinical benefits and safety are still to be determined. The long-term extension trials of PIONEER-HCM and EXPLORER-HCM and post-marketing registries should provide important information that will help define the value of mavacamten for the treatment of oHCM.

Several trials are currently investigating mavacamten in a variety of populations and broader clinical indications. A prospective registry (DISCOVER-HCM; NCT05489705) is assessing the real-world patient characteristics, treatment patterns, and longitudinal outcomes in patients with oHCM treated with mavacamten and other treatments in the US. Another phase 3 trial (HORIZON-HCM; NCT05414175) is underway in Japan to evaluate the effectiveness, safety, and tolerability of a 30-week course and the long-term effects of mavacamten in Japanese patients with symptomatic oHCM. ODYSSEY-HCM is a randomized, double-blind, placebo-controlled, phase 3 trial (NCT05582395) that is evaluating mavacamten in patients with symptomatic non-obstructive hypertrophic cardiomyopathy. Mavacamten is also being investigated in patients who have heart failure with a preserved ejection fraction (HFpEF). EMBARK-HFpEF (NCT04766892) is a phase 2a proof-of-concept study that is assessing the safety, tolerability, and preliminary efficacy of mavacamten treatment on the biomarker levels in patients with HFpEF.

Nevertheless, there are still a lot of unanswered questions. Primarily, mavacamten was not developed to treat LVOT obstruction, but to optimize energetics at the sarcomeric level. Whether it has the potential to limit or even reverse the disease progression, whether it can stop the disease expression in genotype-positive carriers without the HCM phenotype, and whether there are differences in the response depending on the type of genetic mutation, are all unclear and need to be investigated.

Finally, aficamten, a next-in-class cardiac myosin inhibitor has completed a phase 1 assessment, and the data from a phase 2 study showed that treatment with aficamten improved the LVOT gradient, heart failure symptoms and biomarkers, and was well-tolerated in patients with oHCM [48].

## 6. Conclusions

HCM with an LVOT obstruction is associated with increased morbidity and mortality. When standard medication with β-blockers or calcium channel blockers is not sufficient, the next option is SRT. As the first cardiac myosin inhibitor, mavacamten represents a major milestone in the treatment of symptomatic patients with oHCM. The data from phase 2 and 3 trials showed that mavacamten significantly improved the LVOT gradients, biomarkers, and symptoms. Transient reductions in the LVEF were associated with higher plasma drug levels and responded well to dose adjustments. Mavacamten was well-tolerated and the rate of adverse events were low. The data on the long-term safety and efficacy is necessary to determine its place in the treatment algorithm for oHCM alongside the established methods of SRT. Further indications for treatment such as paediatric HCM, non-obstructive HCM, and HFpEF are currently being investigated.

## Figures and Tables

**Table 1:** Summary of the main mavacamten clinical trials.

Study Name	Trial Design	Number of Patients and Treatment	Follow-Up	NYHA Class	Primary Endpoint	Post-Exercise LVOT Gradient	pVO_2_	≥1 NYHA Class Improvement	KCCQ-CCS Score	NT-proBNP	LVEF
						Treatment Difference (95% CI) with *p* Value					
**PIONEER-HCM**	Multicenter, phase II, open-label, nonrandomized. Two sequential cohorts	21 Cohort A: mavacamten 10–20 mg/day alone Cohort B: mavacamten 2–5 mg/day with β-blockers allowed	12 weeks	II/III	Change in postexercise LVOT gradient	Cohort A: −89.5 mmHg (−138.3, −40.7 mmHg) *p* = 0.008 Cohort B: −25.0 mmHg (−47.1, −3.0 mmHg) *p* = 0.020	Cohort A: 3.5 mL/kg/min (1.2, 5.9 mL/kg/min) Cohort B: 1.7 mL/kg/min (0.03, 3.3 mL/kg/min)	N/A	N/A	N/A	Cohort A: −15% (−23%, −6%) Cohort B: −6% (−10%, −1%)
**EXPLORER-HCM**	Multicenter, phase III, randomized, double-blind, placebo-controlled	251 Mavacamten starting at 5 mg/day titrated to 15 mg/day to achieve a <30 mmHg reduction in LVOT gradient and a drug plasma concentration of 350–700 ng/mL	30 weeks	II/III	Clinical response (≥1.5 mL/kg/min increase in pVO_2_ and ≥1 NYHA class reduction, or ≥3 mL/kg/min pVO_2_ increase without NYHA class worsening)	−35.6 (−43.2,−28.1) *p* < 0.0001	1.4 (0.6, 2.1) *p* = 0.0006	34% (22, 45) *p* < 0.0001	9.1 (5.5, 12.7) *p* < 0.0001	0.202 (0.17, 0.24)	−4.0% (−5.5,−2.5)
**VALOR-HCM**	Multicenter, phase III, randomized, double-blind, placebo-controlled	112 Mavacamten starting at 5 mg/day titrated to 15 mg/day based on LVOT gradient and LVEF	16 weeks	III/IV or II with exertional syncope	Eligibility for SRT according to the 2011 guidelines or a patient decision to proceed with SRT	−37.2 (−48.1, −26.2) *p* < 0.001	N/A	41.1 (24.5, 57.7) *p* < 0.001	9.4 (4.9, 14.0) *p* < 0.001	0.33 (0.26, 0.42) *p* < 0.001	−4.0 (−5.5, −2.5)

KCCQ-CCS, Kansas City Cardiomyopathy Questionnaire—symptoms and physical limitation; LVEF, left ventricular ejection fraction; LVOT, left ventricular outflow tract; N/A, not available; NT-proBNP, N-terminal pro B-type natriuretic peptide; NYHA, New York Heart Association; pV0_2_, peak oxygen consumption; SRT, septal reduction therapy.

**Table 2:** Contraindications and adverse reactions of mavacamten.

Contraindications	Adverse Reactions
Drug intolerance	Dizziness
Pregnancy	Syncope
LVEF < 55%	Dyspnea
Concomitant use with a strong CYP2C19 inhibitor and strong CYP3A4 inhibitor	Systolic dysfunction

LVEF, left ventricular ejection fraction.

## Data Availability

Not applicable.

## Abbreviations

ASA	alcohol septal ablation
HCM	hypertrophic cardiomyopathy
oHCM	obstructive hypertrophic cardiomyopathy
LVOT	left ventricular outflow tract
SAM	systolic anterior movement
SRT	septal reduction therapy
SM	septal myectomy
